# “We” Are In *This* Together, But We Are Not One and the Same

**DOI:** 10.1007/s11673-020-10017-8

**Published:** 2020-08-25

**Authors:** R. Braidotti

**Affiliations:** Utrecht University, Achter de Dom 20, 3512 JP Utrecht, The Netherlands

**Keywords:** COVID-19, Anthropocene, Posthumanism, Postcolonialism, Feminism, Intersectionality, Indigenous theories

## Abstract

The COVID-19 pandemic is a man-made disaster, caused by undue interference in the ecological balance and the lives of multiple species. Paradoxically, the contagion has resulted in increased use of technology and digital mediation, as well as enhanced hopes for vaccines and biomedical solutions. It has thereby intensified humans’ reliance on the very high-tech economy of cognitive capitalism that caused the problems in the first place. This combination of ambivalent elements in relation to the Fourth Industrial revolution and the Sixth Extinction is the trademark of the posthuman condition. This essay explores this condition further, offering both critical and affirmative propositions for moving forward.

The COVID-19 pandemic is a man-made disaster, caused by undue interference in the ecological balance and the lives of multiple species. Paradoxically, the contagion has resulted in increased use of technology and digital mediation, as well as enhanced hopes for vaccines and biomedical solutions. It has thereby intensified humans’ reliance on the very high-tech economy of cognitive capitalism that caused the problems in the first place. This combination of ambivalent elements in relation to the Fourth Industrial revolution and the Sixth Extinction is the trademark of the posthuman condition.

The underlying mood during this pandemic is affective. It involves complex and internally contradictory alternation of emotions—that mark what I have called the posthuman convergence (Braidotti 2013, 2019). An intense sense of suffering alternating with hope, fear unfolding alongside resilience, boredom merging into vulnerability. Excitement and exhilaration in view of the advanced technologies that drive the Fourth Industrial Age, flip into anxiety and fear at the thought of the huge costs and damages inflicted by the Sixth great Extinction, on both the human and non-human inhabitants of this planet. Although climate change has come to represent this danger in an almost emblematic manner, and the nuclear threat is far from abated, in the current state of emergency, the centre of all concerns is the COVID-19 pandemic.

Even before the rigours of the lockdown, the closing of the borders and the rising death toll, the intensity and spread of that negative affective economy was palpable. Exhaustion and fatigue—a recurrent sense of hopelessness or impossibility—have become prominent features of the contemporary psychic landscapes, across the urbanized over-developed world. They are witnesses to the daily and nightly struggles to come to terms with what our world has become and the complexities of our historical context.

It is not easy to address these issues. Words, in so many ways, falter and fail. One can only talk about fear, sorrow, and exhaustion in a language reduced to its ossified minimal components, a language that has reached the edge of what it can express, approximating silence but not falling into it just yet. Spiritual fatigue almost longs for a neutralized style that has perfected ways of de-linking from the grand statements of high theory. It would be obscene and unethical to theorize about the epidemiological catastrophe that is unfolding under our very eyes. This is not a time for grandiose theorizing but for collective mourning, affective resistance, and regeneration. We need to mourn the dead, humans and non-humans and not build theories on their dead bodies—that would be a shameless abuse of intellectual power. But over and above all else, we also need to develop different ways of caring, a more transversal, relational ethics that encompasses the non-humans.

There is so much that we need to both embrace and resist: the wave of collective and personal despair at the loss of lives, the hardship of the socio-economic consequences of this man-made disaster, the awareness of all that was wrong with the old world and which has now become manifest. So the first challenge is to find the appropriate language for such an endeavour, a language that remains critical but in a modest capacity. We need to understand and account for the pain of the world, for what this damaged planet is going through, but such knowledge production has to remain de-linked from the pretentions of some sovereign idea of the thinker, the scholar, and the writer as the master signifier. The affective and social climate we are in calls for humility, cooperation, and is antithetical to syntheses and to authoritarian anthropocentric injunctions.

The thoughts that come to mind in attempting to account for our predicament have almost the form of fragments of meditation upon the sorrowful present.

To begin with: viruses born of human interference with animals and environmental sources, such as COVID-19, are anthropogenic and hence discriminate just as much as humans do. They act as indicators of massive social inequalities, which dominant neo-liberal political classes were intent on denying. Now the horrid truth about the consequences of their austerity policies is hitting them in the face: public health is an intensely political issue. If it is undeniable that the “capitalocene”—the greed of consumers’ society—is responsible for the abuse of animal life that produced the infections of the bats and generated COVID-19, it is equally true that neoliberal governance has laid the foundations for the spread of the contagion by exacerbating socio-economic power differences.

Secondly, not all humans are equal and the human is not at all a neutral category. It is rather a normative category that indexes access to privileges and entitlements. Appeals to the “human” are always discriminatory: they create structural distinctions and inequalities among different categories of humans. Humanity is a quality that is distributed according to a hierarchical scale centred on a humanistic idea of Man as the measure of all things. This dominant idea is based on a simple assumption of superiority by a subject that is: masculine, white, Eurocentric, practicing compulsory heterosexuality and reproduction, able-bodied, urbanized, speaking a standard language. This subject is the Man of reason that feminists, anti-racists, black, indigenous postcolonial and ecological activists have been criticizing for decades.

Exposing the power-ridden assumptions of the dominant category of the human also results in relocating the subjects who have come to represent the dialectical opposites and opponents of this dominant and normative vision of the human. These are the less-than-human others, dehumanized or excluded from full humanity—these qualitatively minoritarian subjects actually very often are quantitively the majority. Historically, they have been the sexualized others (women, LBGTQ+); the racialized others (non-Europeans, indigenous); and the naturalized others (animals, plants, the Earth).

In a concomitance of events that marks the extraordinary period we are going through, the voices, experiences, and perspectives of these multiple others are exploding all around us. The power of viral formations has become manifest in the pandemic, stressing the agency of non-human forces and the overall importance of Gaia as a living, symbiotic planet. But at the same time a global revolt again endemic—and indeed viral—racism has also exploded in this fateful June 2020, led by the “Black Lives Matter” movement. As these multiple crises unfold, the politics of the sexualized, racialized, naturalized others are moving centre stage, pushing old Anthtropos off-centre.

Thirdly, it is important to keep in mind that the binary distinctions between nature and culture, humans and non-human have been foundational for European thought since the Enlightenment and that many cultures on earth do not adopt such a partition (Gibson, Rose, and Fincher 2015). This is the strength of the insights and understandings that can be learned from indigenous epistemologies and cosmologies, postcolonial and decolonial thought and African philosophy. Many of them pose a “multinatural” continuum across all species, all of which partake of a distributed idea of humanity. This means that all entities are considered as being endowed with a soul, which assumes a commonly shared human nature that includes the nonhumans. To call this approach “animism,” as colonial powers did, is to miss the point, adding to this ignorance an uncommon dose of epistemic violence. When it comes to human/non-human relations, it is time to start learning from the South.

Fourthly, some critical self-restraint may be needed. A pandemic on the scale of COVID-19, brings home to the Western world an ancient truth: that “we” are all in this planetary condition together, whether we are humans or others. But it is also high time for this heterogeneous and collective “we” to move beyond the Eurocentric humanistic representational habits that have formatted it. Dislodging also the philosophical anthropocentrism they entail and enforce. This shift of perspective inaugurates critical posthuman thought. Nowadays we can no longer start uncritically from the centrality of the human—as Man and as Anthropos—to uphold the old dualities. This acknowledgment, however, does not necessarily throw us into the chaos of non-differentiation, nor does it awaken the spectre of extinction. It rather points in a different direction, towards some other middle-ground, another *milieu*, which expressed the awareness that “we”—all living entities—share the same planetary home.

Yes, we are connected, that is to say ecologically interlinked through the multiple interconnections we share within the nature-culture continuum of our terrestrial *milieu.* But we differ tremendously in terms of our respective locations and access to social and legal entitlements, technologies, safety, prosperity, and good health services. The posthuman subjects of today’s world may be internally fractured, but they are also technologically mediated and globally interlinked. It is important to stress the materially embedded differences in location that separate us but also to stress the shared intimacy with the world that creates a sense of belonging together, within webs of ever-shifting relations.

Fifth insight: feminist theory is of great assistance to think equality with difference, multiple belongings and power rifts, because it stresses the embodied, embedded, and sexed roots of all material entities, humans included, and their unexplored resources. The relevance of feminist thought in times of crisis is to emphasize the multiple perspectives that emerge from attention to embodiment and lived experience. But is also adds an intersectional approach that stresses the inclusion of axes of analysis such as race, age, class, and able-bodiedness. Stressing corporeality, embodiment, and inter-connection is one of the strengths of feminist philosophies, which have replaced discriminatory unitary categories, based on Eurocentric, masculinist, anthropocentric, and heteronormative assumptions, with robust alternatives. The embedded and embodied empiricism at work in feminist theory acts as the source of counter knowledges, methods, and values. They are needed more than ever today.

Sixth insight: post/de-colonial and indigenous theories have a great deal to teach us. Not only do they stress that for most people on earth, the nature-culture distinction does not hold but also that the fear of death and extinction is an integral part of colonized cultures. For many indigenous people on earth, epidemics, dispossession, and environmental devastations were the mark of the colonial conquests and of the Europeans’ appropriation and destruction of First Nations cultures. Catastrophes on this scale are for many people on earth an everyday reality—whether we think of climate change, inter-generational transmission, or public health issues. Europeans have a lot to answer for.

## Political Economy of Affects

In light of these insights, I would reach a preliminary conclusion that we need to be lucid about the affects involved in our current predicament and relativize them a bit as well. We need to resist with equal lucidity the pull of apocalyptic thinking as well as the abyss of self-pity: this is a time to organize, not to agonize. The current crisis can make us more intelligent about what we are ceasing to be and who we are capable of becoming. It enables subtler and more complex cartographies of powers and discourses at work in our societies, that is to say a more adequate rendition of where we are at (Braidotti and Hlavajova 2018). These accounts have to start by questioning who “we” might be to begin with and whose anxiety is taking centre-stage in public debates about the crisis.

Accepting our shared exposure to environmental and public health man-made risks is the starting point for a process of assessing these risks and dealing with them collectively and socially. This approach expresses a sort of epistemological humility that reiterates the never-ending nature of the processes of becoming-humans. An adequate response to a crisis on the scale of COVID-19 calls for community-based, experiments to see how and how fast we can transform the way we live. That means facing up to the negative conditions, the social and environmental inequalities and the collective responsibility towards exposed or vulnerable populations. This praxis of forging communal solutions through the confrontation of uncomfortable truths is central to the ethics of affirmative ethics. It is a praxis that promotes action and critical self-knowledge, by working through negativity and pain. This pro-active activism manifests the living beings’ shared ability to actualize and potentiate different possibilities.

This transformative energy is the core of affirmative ethics, which stresses the inexhaustible potential of all living organisms—humans and non-humans—to generate multiple and yet unexplored interconnections. This is the immanence of a life that can only be co-constructed and jointly articulated in a common world. What is inexhaustible is not some transcendental and abstract notion of Life with capital letters but rather the more patient task of socially co-constructing one’s life, alongside so many others.

Just one life, following the formula of the ancient Stoics, can only be predicated in a constant, friendly companionship with pain and suffering. This in turn means that ethics is the practice of extracting knowledge and wisdom from the reworking of pain. Pushed to the extreme, it brings us face to face with mortality, the extreme manifestation of vulnerability. Death is the painful event par excellence, but it is also the event that marks our inscription into the time of our life. We need to make friends with death. At the level of awareness, it is the event that has always already happened, because to be born means to become mortal. As such, it is a strangely impersonal event. Death marks the outer boundary of the limited time we have at our disposal. Being aware of this limit can be an energizing thought, not a catastrophe. Affirmative ethics encourages us to train for making the most of one’s powers and capabilities, so as to become the most affirmative possible version of what one could be, through the pain and the acknowledgment of mortality.

Posthuman resistance must mobilize for the compositions and collective construction of affirmative forms of action and solidarity and can activate their own generative force. Many lives today are the object of biopower’s thanato-politics, doomed to ethnic cleansing or slaughter, to being killed without their killer being held accountable. Think of the refugees dying on the edges of Fortress Europe or on Manus Island. We are vulnerable to viruses and other illnesses, to the effects of climate change and other devastations—many of these exposed lives are not human. But our shared life as an inhuman, non-anthropocentric force (which I call *zoe*)—exceeds these negative conditions, because *zoe* exists independently of humans.

Many of us are struck about how, in the middle of this pandemic, Spring is advancing, flowers are blooming, the earth keeps on growing—regardless.

Humans are not the centre of creation. This is the greatness of affirmative thought as a secular, materialist philosophy of becoming. It is an inexhaustible generative force that potentially can transmute lives into sites of resistance—all lives, also the non-human. Life is a generative force beneath, below, and beyond what we humans have made of it. *Zoe*/geo/techno-perspectives at the core of this heterogeneous definition of life are sites of resistance. They provide multiple alternatives to the devastations of necropolitics and the entrapment of biopolitical management of Life as capital.

But what a huge task that is! Fatigue, fear, and despair overlap and accumulate to produce a feeling of utter impotence. This closing down of the horizon of possible actions is the symptom of the negativity of our times. Negativity expresses itself in a social and psychological dimming of a sense of possibility, which triggers a systemic fragmentation and a shattering of our relational capacity. This weakening of the desire to act often feeds an appeal to external powers to take over the task of organizing how to live our lives. This negativity ultimately brings about a shrinking of our ability to take *in* and *on* the world that we are in, simply because it hurts too much to take it in and on. We have to dose how much of it we can take, till it gets too much. Too-much-ness is one of the sources of exhaustion, which marks so much of or current predicament.

What is inexhaustible, however, is our desire to persevere in living, against all odds. This is the innermost essence or *potentia* of all living entities: the life in me that does not answer to my name. This vital sense of life is not to be taken for granted or to be sacralized in religious terms. It remains materialist and secular. “Just a life” expresses a deep sense of belonging to a common world, the one world we have in common. The desire to get on with it, is the fragile yet irrepressible bond that interconnects all living entities. This produces a roar of energy that is mostly unperceived and imperceptible, yet indispensable.

What is inexhaustible is our capacity, our power even, to differ within ourselves, as well as between us. We can extract ourselves from this sad state of affairs, work through the multiple layers of our exhaustion, and co-construct different platforms of becoming. This transformative praxis can only be enacted collectively, together, as transversal subjects of posthuman times. Shared exhaustion actually unfolds upon a deeper wisdom about what it is exactly that one knows, when one is facing momentous changes in unfamiliar territories. One knows that Life lives on regardless of human pretensions and expectations. “We” can only intervene in *this* as transversal ensembles, acting collectively: “We”-who-are-not-one-and-the-same-but-are-in-this-convergence-together.

Melbourne artist Patricia Piccinini (2020) made this point clearly with her new public art campaign about the bat-boy

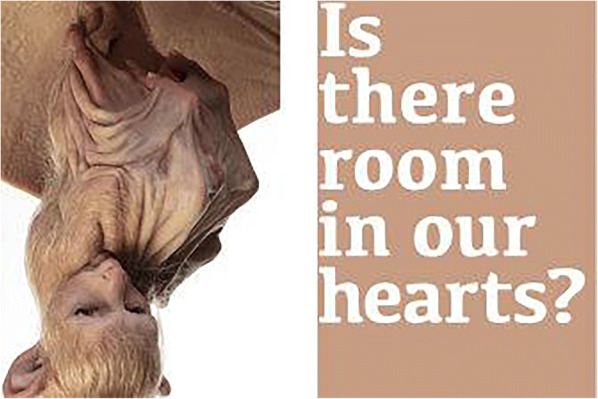



Are we capable of becoming this kind of posthuman caring hearts? Are we prepared to steer this path together? To become a “we”—the missing people?
